# Pharmacogenetics: polymorphism and genotype-phenotype correlation of drug response in indian population

**DOI:** 10.1186/1755-8166-7-S1-I52

**Published:** 2014-01-21

**Authors:** Harish Padh

**Affiliations:** 1 Sardar Patel University, Vallabh Vidyanagar, Gujarat, India

## 

Inter-individual genomic variations have recently become evident with advances in sequencing technologies. Polymorphism and human variants have been linked to disease susceptibility as well as drug efficacy and toxicity. Although polymorphism can occur at any loci and can affect any protein structure and function, it is largely studied in enzymes involved in drug metabolism where much correlation has been established with drug efficacy and toxicity. Among genetic variations SNPs are widely studied and better defined because of availability of large scale detection platforms. Besides SNP, insertion-deletions (INDELs), inversions, copy number variations (CNVs) and larger structural variations (> 3 Mb) have come to light in recent years, however, their link to health and disease remain ill-defined. CNVs are defined as the segment of DNA larger than 1 Kb in size, and compared to reference genome vary in copy number.

All types of genomic variations are bound to play vital role in disease susceptibility and drug response. In this presentation, genetic variation in many DMEs like CYP2D6, CYP2C9, CYP2C19 will be discussed, and its effect on pharmacokinetic (PK) parameters like AUC, Cmax, Tmax and T1/2 will be presented. PK variation as phenotype will be compared and correlated with genotype variation in Indian population. Examples of CNV data in Indian population will be presented and compared with other populations. Available literature will pose significant policy issues about drug approval procedure. The issue of incorporation of local pharmacogenetic consideration in drug approval will be analysed.

